# Different Migration Patterns of Sea Urchin and Mouse Sperm Revealed by a Microfluidic Chemotaxis Device

**DOI:** 10.1371/journal.pone.0060587

**Published:** 2013-04-16

**Authors:** Haixin Chang, Beum Jun Kim, Yoon Soo Kim, Susan S. Suarez, Mingming Wu

**Affiliations:** 1 Department of Biomedical Sciences, Cornell University, Ithaca, New York, United States of America; 2 Department of Biological and Environmental Engineering, Cornell University, Ithaca, New York, United States of America; National Research Council, Italy

## Abstract

Chemotaxis refers to a process whereby cells move up or down a chemical gradient. Sperm chemotaxis is known to be a strategy exploited by marine invertebrates such as sea urchins to reach eggs efficiently in moving water. Less is understood about how or whether chemotaxis is used by mammalian sperm to reach eggs, where fertilization takes place within the confinement of a reproductive tract. In this report, we quantitatively assessed sea urchin and mouse sperm chemotaxis using a recently developed microfluidic model and high-speed imaging. Results demonstrated that sea urchin *Arbacia punctulata* sperm were chemotactic toward the peptide resact with high chemotactic sensitivity, with an average velocity *V_x_* up the chemical gradient as high as 20% of its average speed (238 μm/s), while mouse sperm displayed no statistically significant chemotactic behavior in progesterone gradients, which had been proposed to guide mammalian sperm toward eggs. This work demonstrates the validity of a microfluidic model for quantitative sperm chemotaxis studies, and reveals a biological insight that chemotaxis up a progesterone gradient may not be a universal strategy for mammalian sperm to reach eggs.

## Introduction

Chemotaxis is a widespread natural phenomenon in which cells or organisms guide their movements according to a chemical gradient in their environment. Chemotaxis is critical in various cellular activities [1], including food searching by bacteria [2], immunological responses [3], and cancer cell migration during metastasis [4]. Sperm chemotaxis, where sperm are guided toward eggs by their secretions, has been demonstrated to occur in some species [5], [6]; however, it is not clear if chemotaxis is a universal strategy for sperm of all species to find eggs.

It has been recognized that chemotaxis of sperm toward eggs plays an important role in fertilization in some marine invertebrate species. The gametes of marine invertebrates such as the purple sea urchin (*Arbacia punctulata*) and the red abalone (*Haliotus rufescens*) [7] have served as model systems for sperm chemotaxis studies. The marine invertebrates of many species are external fertilizers; that is, they broadcast their sperm and eggs into the surrounding water, which is often moving in nature. Under these conditions, specificity of chemoattractants for conspecific sperm is high in order to avoid interspecies fertilization [7], [8] while promoting successful encounters between the sperm and the eggs in moving water. In the sea urchin species *Arbacia punctulata,* sperm are specifically attracted to resact, a small linear polypeptide that diffuses out from the egg jelly coat [9]. *Arbacia* sperm are extremely sensitive to resact and respond to a broad range of resact concentrations. It is estimated that they can respond to as little as a single molecule of resact and to over six orders of magnitude of concentrations [10]. The response of *Arbacia* sperm involves a ‘‘turn-and-run’’ pattern, in which the sperm swimming paths alternate rapidly between slightly curved paths (run) and deeply curved paths (turn). The turn-and-run pattern is regulated by Ca^2+^ signaling [6].

There is some evidence to support the existence of sperm chemotaxis in mammalian fertilization. One fundamental difference between mammalian and *Arbacia* sperm is that mammals are internal fertilizers, where sperm migrate toward the egg in the physically constrained environment of the female reproductive tract. In humans, it has been reported that ovarian follicular fluid [11], [12], [13], [14], [15] or medium conditioned by the cumulus cells that surround the oocyte [16], [17] attracts sperm. Progesterone was proposed to be the primary active agent in follicular fluid [15], [18] and cumulus cell secretions [19]; therefore, it is plausible that a gradient of progesterone could form within and surrounding the cumulus mass after the egg enters the oviduct. Progesterone gradients in pico-molar ranges or up to micro-molar levels have been reported to produce chemotaxis in human and rabbit sperm. In those studies, the progesterone gradients were generated in Zigmond (or Dunn) chambers and the conclusions were chiefly based on calculating the percent of sperm with tracks whose orientation is less than 45 degrees with respect to the direction of the progesterone gradient [19], . The progesterone concentration fields in these experiments were largely unknown.

Recent developments in microfluidic technology have opened up opportunities to quantitative studies of cellular chemotaxis [22], particularly because of the opportunities provided to create well-defined chemical gradients and enable single cell analysis. In this article, we present and compare side-by-side the quantitative analysis of sperm movement pattern of two model species, sea urchin (*Arbacia punctulata*, an external fertilizer) and mouse (*Mus musculus,* an internal fertilizer) in the presence of gradients of putative chemotactic agents. We use a microfluidic model to generate well-defined gradients and live cell imaging to follow sperm movement both in time and space.

## Results

### Microfluidic device setup and cell migration characterization

**Figure 1:**
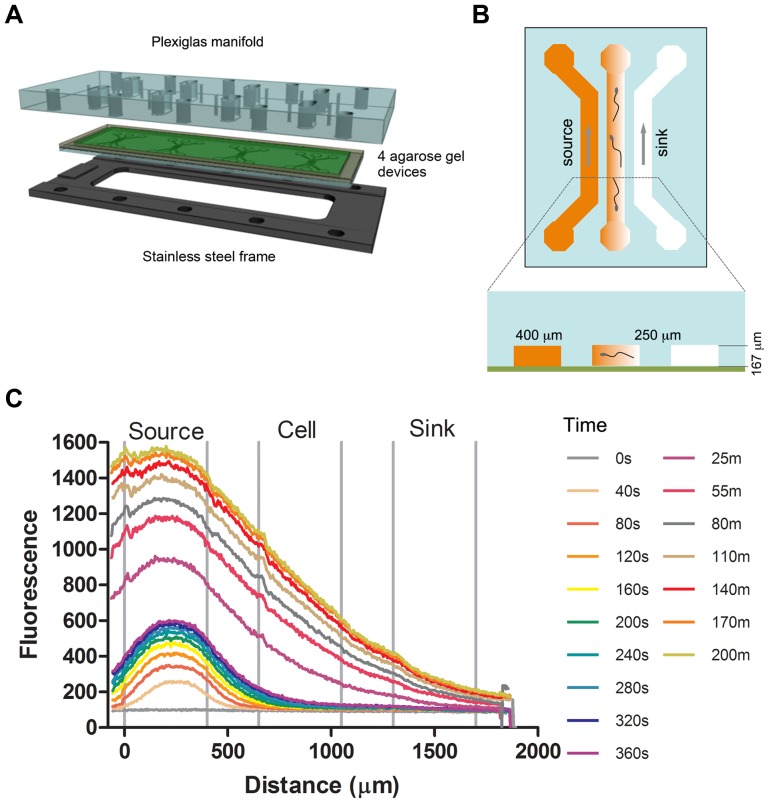
Microfluidic device setup, operation principle and calibration. (A) Device setup. Four devices were patterned on a 1 mm thick agarose gel membrane, which was sandwiched between a Plexiglas manifold and a stainless steel supporting frame (Drawing credit: Andrew Darling). (B) Device layout. Each device contained three parallel channels that were 400 µm wide and 167 µm deep, and spaced 250 µm apart. Sperm are not shown to scale. This drawing is reproduced from Ref. [45] by permission of The Royal Society of Chemistry. Chemical/buffer were flowed through two side channels and a chemical gradient formed in the center channel via molecular diffusion through the agarose ridges between the center and the side channels. (C) Device characterization. Time evolution of fluorescence intensity profile across all three channels when flowing 4 kDa FITC-dextran/buffer along the source and sink channels respectively. Time (t)  = 0 is defined as the time when the chemoattractant was flowed into the source channel.

Chemical gradients were generated using a recently developed microfluidic device (See Fig. 1 and Fig. S1) [3], [23]. Briefly, three parallel microfluidic channels were patterned on a 1 mm thick agarose gel membrane. Sperm were seeded into the center channel prior to gradient formation. Species-specific buffered medium, with and without chemoattractant, were then introduced into the two side channels, the source and sink channel, respectively, to form a chemical gradient across the center channel via molecular diffusion. Time (t)  = 0 is defined as the time that the flows of chemoattractant and media were started through the source and sink channels. This device has been characterized for its ability to generate steady and well-defined gradients, both numerically and experimentally, using a COMSOL multi-physics software (COMSOL Inc., Burlington, MA) and FITC-conjugated proteins [3], [23]. Fig. 1C shows calibration curves obtained by running FITC-dextran (4 kDa) dissolved in artificial sea water (ASW) through the source channel and ASW through the sink channel, and demonstrates that the gradient reaches a steady state in about 25 minutes. Comparison to numerical simulation can be found in Fig. S2.

### Contrasts in the morphology and motility between sea urchin and mouse sperm

**Figure 2:**
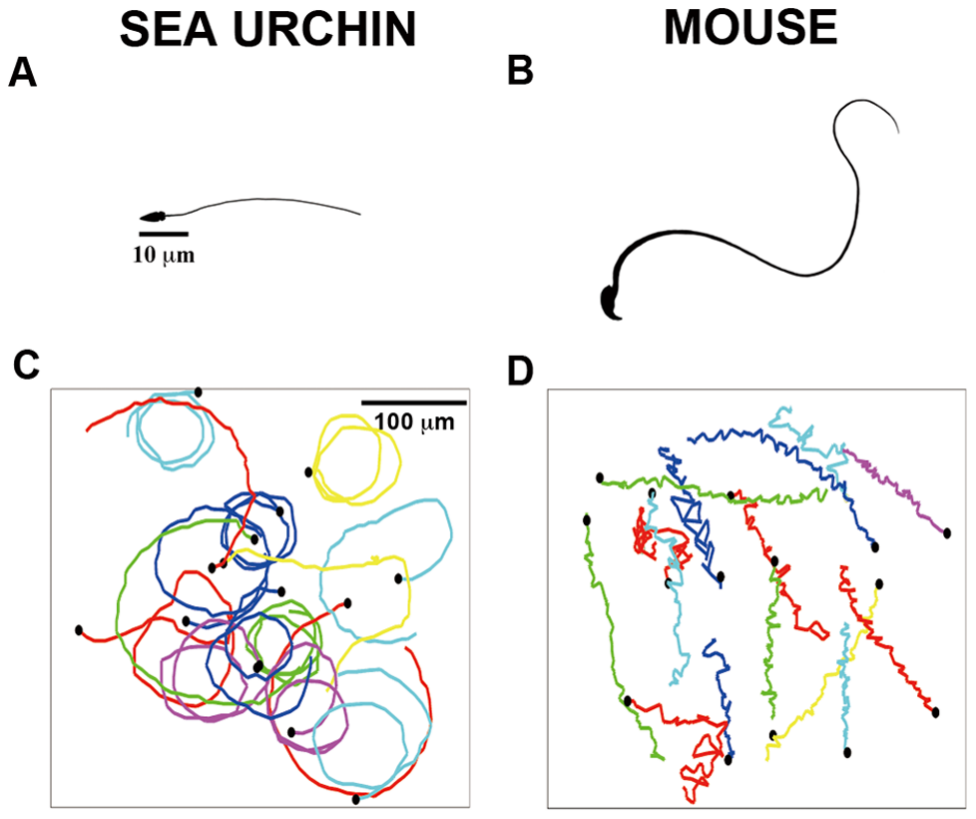
Differential morphology and motility of sea urchin versus mouse sperm. An illustration of sea urchin (A) and mouse (B) sperm drawn to scale. Both sea urchin and mouse sperm use flagella having axonemes of 9 outer microtubule doublets and a single central pair of microtubules in order to move. Sea urchin sperm has a typical length scale of 50 µm, and mouse sperm 100 µm. Drawing credit: C. Rose Gottlieb. Trajectories of sea urchin (C) and mouse (D) sperm swimming in a microfluidic channel in the absence of putative chemoattractant. Each colored line represents a trajectory, and each trajectory is 2 s long and starts at t = 0.

Fig. 2A shows that the sea urchin sperm has a cone-shaped head and is about 60 μm in length. In control medium, sea urchin sperm swim in a circular trajectory by propagating asymmetrical waves down the flagellum. The diameter of the circles is similar to their body length (Fig. 2C, also see Movie S1). Fig. 2B (also Movie S2) shows that the mouse sperm, on the other hand, has a hook-shaped head, and is about 120 μm in length. The motor apparatus (axoneme) in the core of the flagellum of mouse sperm is structurally identical to that of sea urchin sperm; however, the mouse sperm motor apparatus is surrounded by additional skeletal elements (outer dense fibers and fibrous sheath) that affect the shape of the flagellar wave as it forms and propagates down the tail.

When male mice inseminate females, the sperm begin swimming in a linear trajectory that results from nearly symmetrical flagellar beating and continuous rolling about the long axis. However, before mouse sperm approach the site of fertilization in the oviduct, they switch to a swimming pattern in which the asymmetry of the flagellar beat pattern increases and the sperm cease rolling but often flip on their longitudinal axis [24] (Fig. 2D, also see Movie S2). This asymmetrical flagellar beating pattern is referred to as “hyperactivation”.

### Contrasting swimming behavior of mouse and sea urchin sperm in chemotactic gradients

**Figure 3:**
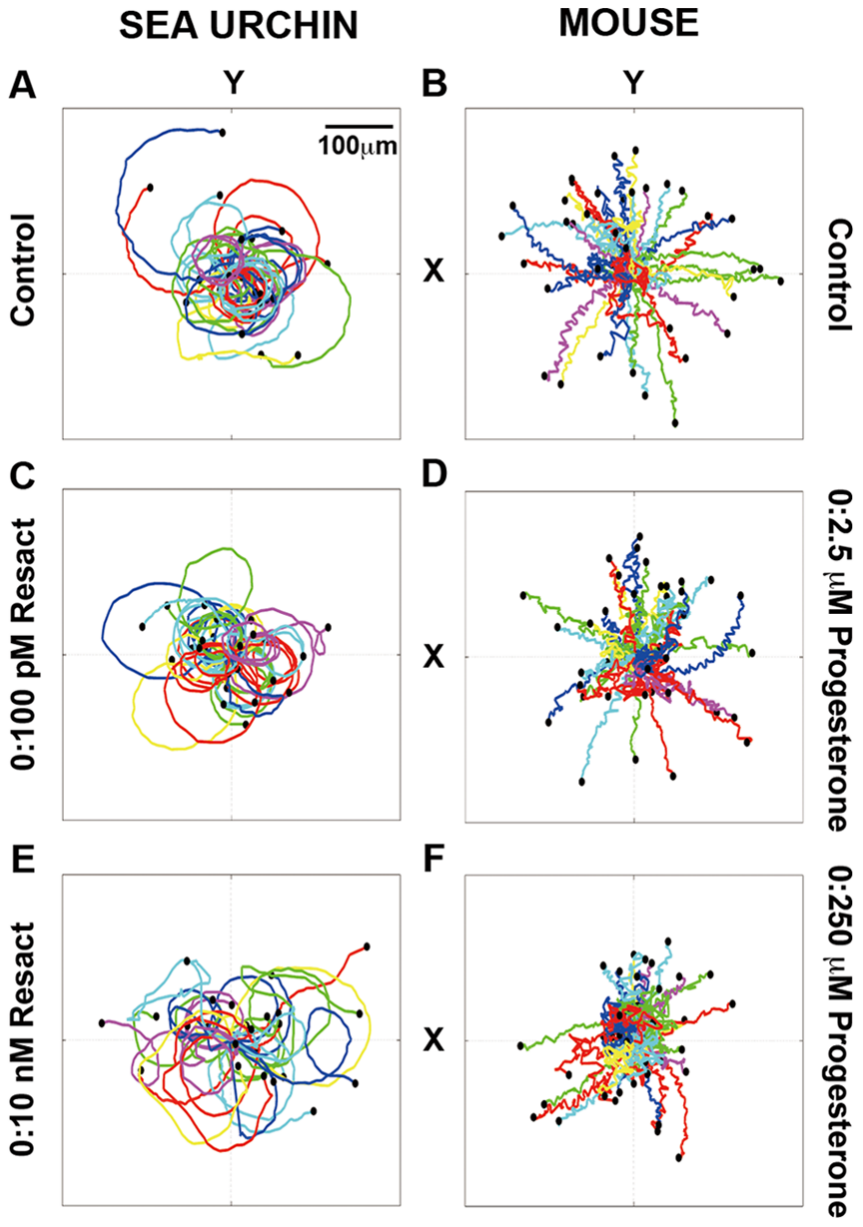
Differential chemotactic behavior of sea urchin and mouse sperm. Trajectories of sea urchin sperm (25 sperm in each plot) when the resact concentration in the source channel is 0 (A); 100 pM (C); 10 nM (E). Trajectories of mouse sperm (39 sperm in each plot) when the progesterone concentration in the source channel is 0 (B); 2.5 µM (D); 250 µM (F). We placed the starting point of each of the trajectories at the (0, 0) coordinate. Each colored line is a cell trajectory that is 2 s long and starts at t = 0.

Dynamics of mouse and sea urchin sperm swimming were recorded as soon as the sperm were added and subsequent fluid disturbance settled in the center channel of the microfluidic device, which was when the generation of the chemotactic gradients was begun. At 8×10^6^/ml of sea urchin sperm (low cell density) and 10×10^6^/ml mouse sperm, individual sperm from both species could be followed and tracked (See Movies S3 and S4; Fig. 3). Polar plots were made of re-constructed sperm tracks to show directional motion; the starting point of each track was placed at the origin of the plots, or (0, 0) coordinates. Under control conditions, no obvious chemotactic behavior was observed for either sea urchin or mouse sperm (Fig. 3A and B, also see Movies S1 and S2). Under gradient conditions, clear chemotactic motion along +x direction (the direction of increasing chemoattractant concentration) was observed for sea urchin sperm (Fig. 3C and E, also see Movie S3). However, mouse sperm did not show a clear directed motion in either of the gradients tested (Fig. 3D and F, see also Movie S4).

These results are consistent with the set of experiments when high sea urchin sperm density was used (120×10^6^ ml). Movie S5 shows that sea urchin sperm swim rapidly along the resact gradient. When the source and sink channels were switched, the sea urchin sperm also switched direction to swim toward the resact source. In contrast, the mouse sperm in the center channel remained evenly distributed in the presence (Movie S4) or absence (Movie S2) of a progesterone gradient. This result was also observed when using high-density mouse sperm (data not shown).

### Quantitative analysis of sperm chemokinesis and chemotaxis in the presence of chemoattractant gradients

**Figure 4:**
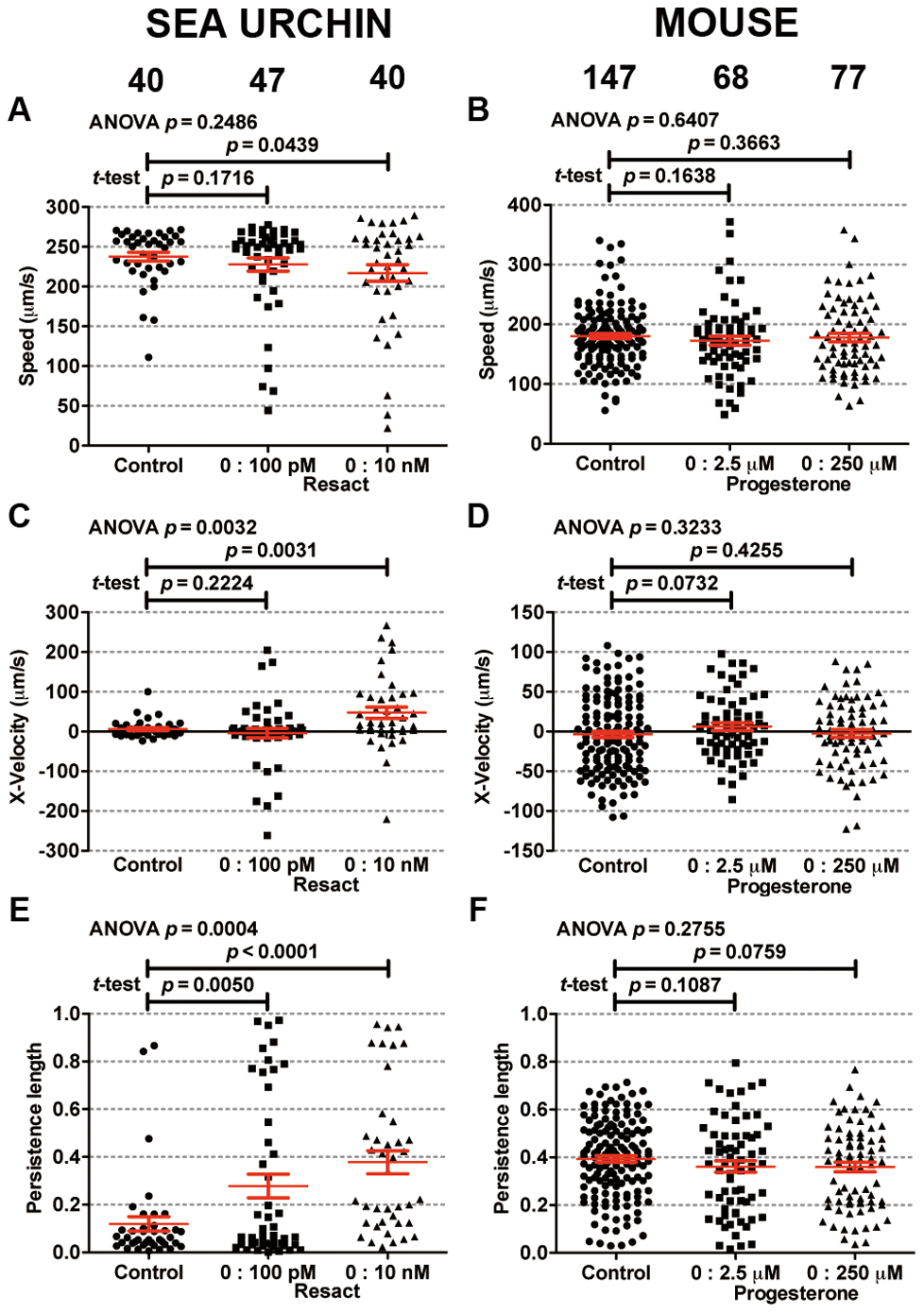
Quantitative analysis of sea urchin and mouse sperm migration pattern. Scatter plot of the speed of sea urchin (A) and mouse (B) sperm. Scatter plot of the velocity up the chemical gradient for sea urchin (C) and mouse (D) sperm. Scatter plot of the persistence length for sea urchin (E) and mouse (F) sperm. Cell numbers that contribute to the scatter plot are indicated. The duration of the cell track length ranges from 0.24 s to 38.0 s for sea urchin, and from 0.12 s to 13.76 s for mouse sperm.

Both sea urchin and mouse sperm did not display chemokinesis, because their speeds remained constant when subjected to chemoattractant gradients as shown in Fig. 4A and B. In the absence of putative chemoattractant gradients, the measured average swimming speed of sea urchin sperm is 238±6 μm/s, which is consistent with the value previously reported for sea urchin (218±30 μm/s; [10]). For mouse sperm, we measured an average swimming speed of 180.4 μm/s, which was similar to 190 μm/s reported elsewhere [25].

The computed average sperm swimming velocity along the putative chemical gradients demonstrates a statistically significant chemotactic behavior for sea urchin (*p* = 0.0032 with one-way ANOVA) but not for mouse sperm (*p* = 0.3233). In our experiments, 100 pM or 10 nM resact were pumped in the source channel and medium in the sink channel. Individual sea urchin sperm were tracked for up to 38 s, which was the estimated time that most sperm took to cross the channel. The resact concentration profiles changed with time during the first 38 s, which are shown in Fig. S3A and Fig. S3B. The maximum resact concentration in the channel during this time period was 2.4 pM (for the case of 100 pM in source channel) or 240 pM (10 nM in source channel). The chemotactic behavior was observed in the case when 10 nM resact (or a maximum of 240 pM resact in the center channel) were pumped in source channel (Fig. 4C, *p* = 0.0031 with *t*-test). The ratio of the average chemotactic velocity and average speed *V_x_*/*U* was 0.22, which is comparable to the value of 0.22 for dendritic cell chemotaxis in chemokine CCL19 gradient [3]. The measurements of the persistence length were consistent with the average velocity measurements (Fig. 4E). It should be noted that the measured resact concentration at which a chemotactic response was observed is higher than the reported resact binding constant. Resact is known to have extremely high binding affinity to its receptor: at *K*_D_  = 1.6±0.6 pM, resact induces an intracellular Ca^2+^ increase [10].

For mouse sperm, 2.5 μM or 250 μM progesterone were pumped into the source channel and medium into the sink channel respectively. Individual mouse sperm were tracked for up to 14 s, which was the estimated time that most sperm took to cross the channel. This is less than the amount of time (38 s) that sea urchin sperm were tracked, because mouse sperm trajectories were straighter and it took less time for them to reach a wall. The computed progesterone concentration profiles during the first 14 s are shown in Fig. S3C and Fig. S3D. The maximum progesterone concentration in the central portion of the center channel reached 8.1 nM, or 820 nM for the case when 2.5 μM or 250 μM progesterone were pumped into the source channel respectively. In all cases, the measured average sperm velocity or persistence length along the gradient direction did not show a statistical difference when compared to control group (*p*-values from a *t*-test for speed, velocity, and persistence length when 2.5 μM progesterone were pumped were 0.1638, 0.0732, and 0.1087, respectively; those when 250 μM progesterone were pumped were 0.3663, 0.4255, and 0.0759, respectively). Thus, no chemotactic behavior was detected (See Fig. 4D and F). For comparison, it was reported that the progesterone promoted a calcium response in human sperm at *K*_D_  = 7.7±1.8 nM [26], which is within the range of our experiments: (0, 8.1 nM) and (0, 820 nM).

## Discussion

To our surprise, our findings revealed that progesterone may not be a universal chemoattractant for mammalian sperm. Extensive efforts were made to test a range of physiological conditions of mouse sperm (collected from epididymis, collected from uterus after mating, non-capacitated, and capacitated) and a wide range of progesterone concentrations. We argue that the differential migration patterns observed here of sea urchin (*Arbacia)* and mouse sperm in chemoattractant gradients correlate with the different fertilization systems used by the two species. Sea urchins use external fertilization, in which about 10 billion sperm and 50,000 eggs are released into the surrounding water [27], [28] and sperm must reach the eggs before sperm and eggs are dispersed by currents. Mice and other male mammals inseminate millions of sperm into the female in order to fertilize a small number of eggs, which is a similar sperm:egg ratio as in the case of sea urchins; however, mammalian sperm are inseminated into a tubular reproductive tract that may confine and regulate sperm migration. In the case of sea urchins, suppose that the ocean current has a velocity of 1 cm/s [7] (corresponding to a Reynolds number of 100 for a length scale of 1 cm, distance between the egg and the sperm). We find that a sea urchin sperm will need to have 1 s of response time to reach an egg before it is swept away by the ocean current (here we use water viscosity of 1 cs for this calculation [7]). This is consistent with our observation shown in Fig. 3E and Movie S5 where the response time of the sea urchin sperm is ∼2 s. For mouse sperm, the distance from the uterus to the site of fertilization in the oviductal ampulla is ∼14 mm [29]. Assuming that the mouse sperm is doing a one dimensional random walk with a speed *v* ∼116 μm/s (measured from movies of sperm in the oviduct taking into consideration of the viscosity of the oviduct fluid and stickiness of the walls [29], with a characteristic time of τ ∼2 s (this is the lower end of the estimate from Fig. 2), then the diffusion constant of the one dimensional random walk is *D* = *v* ^2^ τ = 0.0269 mm^2^/s. The upper limit for time to reach a distance of 14 mm will be *l* ^2^/2D  = 3.64×10^3^ s (61 min). Timed mating experiments with periovulatory female mice indicate that the fastest time for sperm to reach the site of fertilization from the uterus is ∼1 h [30], [31]; therefore, random walk could account for fertilization in the mouse and chemotaxis would not be required.

Although we did not detect a chemotactic response of mouse sperm to progesterone, our results cannot completely rule out the possibility that chemotaxis plays a role during fertilization in mice. Another chemoattractant may be involved, or additional chemical ligands may be required. Furthermore, if chemotaxis in the fluid environment of the oviduct does not exist, sperm migration may still be controlled by other physical or chemical guiding mechanisms. For example, we recently reported that mouse sperm repeatedly attach to and detach from the epithelium that lines the oviduct as they move up to the site of fertilization [29], indicating that there could be a solid phase chemical guidance mechanism involving receptors on the surface of the oviductal epithelium. There is evidence for specific ligand/receptor interactions between sperm and oviductal epithelium in species such as hamsters [32], pigs [33] and cattle [34], [35].

This work demonstrates the validity of microfluidic device for sperm chemotaxis experiments. Our microfluidic model enables a quantitative analysis of sperm movement in a defined and reproducible chemical gradient. In previous reports of progesterone attracting mammalian sperm, there was evidence that the chemotactic responses are weak in comparison to those of sea urchin sperm. First of all, very low percentages of mammalian sperm (1–12%) were reported to orient along gradients of progesterone. Conclusions were based on complex statistical analysis of the orientations of the sperm tracks [13], [36], [37], and there was no direct visualization of the spatial re-distributions of the sperm density in the presence of chemoattractant gradients. However, there were no distinct outliers in either x-velocity or persistence length in our scatter plots (Fig. 4D and F), which reduces this possibility at the given conditions (below 1 out of 68 or 77). In contrast, in sea urchin sperm a robust response can be directly observed (See Movie S5). Secondly, progesterone has been reported to have various effects on sperm. Concentrations from μM to mM progesterone have been shown to induce acrosome reactions in mouse and human sperm [38], [39], and also to prime sperm to undergo acrosome reactions in response to zona pellucida proteins [39], [40], [41]. When sperm undergo the acrosome reaction, the acrosomal region of the head becomes particularly sticky and could thereby cause sperm to accumulate at the source of a progesterone gradient. Under these circumstances, sperm accumulation cannot be differentiated from chemotaxis. The microfluidics device used in our experiments avoids the problem of accumulation of sperm, particularly because sperm do not bind to agarose; therefore, it might be useful to test human and rabbit sperm in the device.

## Materials and Methods

### Media and Chemicals

All routine chemicals and compounds were purchased from Sigma-Aldrich Co. with exceptions noted below. ASW medium was used to activate and dilute dry sea urchin *Arbacia punctulata* sperm. The artificial sea water was composed of 423 mM NaCl, 9.27 mM CaCl_2_, 9 mM KCl, 22.94 mM MgCl_2_, 25.5 mM MgSO_4_, 0.1 mM EDTA, 10 mM HEPES, with pH adjusted to 7.8 by NaOH [10]. For sea urchin sperm chemotaxis experiments, resact (Phoenix Pharmaceuticals Inc., Balmont, CA) was diluted with ASW to the desired concentrations. Mouse sperm capacitation medium [42] consisted of 110 mM NaCl, 2.68 mM KCl, 0.36 mM NaH_2_PO_4_, 25 mM NaHCO_3_, 25 mM HEPES (EMD Chemicals, Gibbstown, NJ), 5.56 mM glucose, 1.0 mM pyruvic acid, 0.006% penicillin G(Na), 2.4 mM CaCl_2_, 0.49 mM MgCl_2,_ and 5 mg/ml BSA (EMD Chemicals). The medium was adjusted to pH 7.6 and 290–310 mOsm/kg and sterilized by filtration through a 0.22 µm membrane filter (Corning Inc., Corning, NY). Before use, it was equilibrated at 37°C with 5% CO_2_ in humidified air. For mouse sperm chemotaxis experiments, progesterone was dissolved in anhydrous DMSO as a stock solution and diluted with mouse sperm capacitation medium to the desired concentrations right before use.

### Animals and sperm collection

Sea urchins *Arbacia punctulata* were purchased from Gulf Specimen Marine Laboratory (Panacea, FL) and kept in an ASW tank (salinity 33–36 p.p.t. and temperature 18–20.5°C). To obtain sperm, individual sea urchins were selected randomly and 0.2 ml of 0.5 M KCl was injected in the coelomic cavity. Sperm were collected from the male animals in air using a pipette. Collected sperm (referred to as dry sperm) were stored at 4°C and used within 2 days.

CD-1 mice aged 8 to 16 weeks were purchased from Charles River Laboratories, MA. The Institutional Animal Care and Use Committee at Cornell University approved all procedures (protocol number: 2009-0011). Ejaculated sperm were obtained from the uteri of mated, superovulated females. Female mice were superovulated by injection with 10 IU PMSG (VWR, Radnor, PA) followed by 10 IU hCG (VWR) 48 h later [43]. Matings were timed to occur 12–14 h after hCG injection. To mate mice, a single hormonally stimulated female was introduced to the cage of a singly housed male. Time of ejaculation was recorded when the male suddenly and briefly became very still while grasping the female. Thirty minutes after mating, the females were euthanized by CO_2_ inhalation to obtain access to the uterine horns, and all efforts were made to minimize suffering. The ejaculated mouse sperm were flushed out of the uterine horns using mouse sperm capacitation medium. Recovered sperm were incubated in an Eppendorf tube at 37°C, 5% CO_2_ in humidified air for 10 min and then the upper layer, which contained highly motile sperm, was collected. Sperm concentration was adjusted with mouse sperm capacitation medium to 10×10^6^ /ml and sperm were incubated for 2 h at 37°C, 5% CO_2_ in humidified air to enable capacitation. The term “capacitation” is defined as changes undergone by sperm in order to be capable of fertilizing eggs. There is evidence that mammalian sperm must be capacitated in order to respond the chemotactic signals [13].

### Microfluidic device setup

The microfluidic chemotaxis device was assembled as previously described [44]. Briefly, a 3% (w/v) agarose solution was made by dissolving agarose (Fisher Scientific, NH) in ASW or BSA-free mouse sperm medium. The solution was then poured onto the area surrounded by the PDMS ring spacer on the silicon master. The solution was polymerized at room temperature while being pressed down by a glass slide. The agarose gel membrane was then rinsed with ASW or BSA-free medium and sandwiched between a glass slide and a Plexiglas manifold with the channel side towards the glass slide. The entire sandwich was secured to a stainless steel support with screws. Fig. 1A shows schematics of the device.

### Gradient profile calibration

Both fluorescein (332 Da) and FITC-dextran (4 kDa) dissolved individually in ASW, and fluorescein dissolved in mouse sperm medium were used for visualizing the concentration gradients established in the center channel. Note that the molecular masses of progesterone and resact are 314 Da and 1245 Da respectively. Prior to the start of an experiment, the channels in the assembled device were loaded with ASW or mouse sperm medium for at least 30 min and then washed with ASW or mouse sperm medium before use. For estimating resact concentration profiles, experiments were conducted at room temperature. For estimating progesterone concentration profiles, the experiments were conducted in a 37°C environment. The tubing used to transport fluorescein dissolved in mouse sperm medium was flushed with the same solution for at least 1 h prior to the experiment.

Fluorescent images of the center channels were taken when either FITC-dextran or fluorescein solutions were pumped through two side channels at a rate of 2 µl/min by a syringe pump (KDS-230, KdScientific, MA). The time t = 0 was set as the time point when the pump started to pump.

Experiments were repeated 3 times for each calibration.

### Chemotaxis assays

Prior to the start of an experiment, the channels in the assembled device were loaded with ASW or mouse sperm medium for at least 30 min and then washed with ASW or mouse sperm medium before use. For sea urchin sperm, experiments were conducted at room temperature. For mouse sperm, the experiments were conducted at 37°C, using pre-warmed devices and fluids. The tubing used to transport progesterone solutions was flushed with the same solution for at least 1 h prior to the experiment to allow BSA in the medium to block any progesterone binding sites in the tubing.

For sea urchin sperm chemotaxis, the dry sperm were diluted with ASW to the desired concentration and the sperm suspension was pipetted into the central channel of the assembled device. The resulting flow in the central channel was stopped by plugging the end of reservoirs. Then, the resact solution and ASW were pumped into the source and sink channels, respectively, at 2 µl/min by the syringe pump. In control devices, ASW was pumped into both the source and sink channels. The source and sink channels were chosen randomly to eliminate experimental bias. The moment when the solutions were pumped into channels was set to be t = 0. To visualize the massive sea urchin sperm migration (high cell density), a 120×10^6^ /ml sperm suspension was loaded into the central channel and 25 or 250 nM resact solutions were pumped into the source channel. In order to track individual sperm, an 8×10^6^ /ml sperm suspension (low cell density) was loaded into the central channel, while 100 pM and 10 nM resact solutions were pumped into the source channels.

For mouse sperm chemotaxis, since mouse sperm are very fragile, the 10×10^6^ /ml sperm suspensions were loaded into the central channel by gently pipetting 10 µl sperm suspension at one end and 5 µl at the other end. This created a pressure difference between the two ends of the center channel, and gentle flow driven by gravity caused the sperm to fill the center channel. The flow in the central channel was then stopped by plugging the end of reservoirs. Then, the 2.5 or 250 µM progesterone solution and control medium (which contained the same amount of DMSO solvent as 2.5 or 250 µM progesterone, 0.008 and 0.8% respectively) were pumped into the sink and source channels respectively by the syringe pump. As a control, medium containing the 0.8% DMSO was pumped into both of the source and sink channels of another device.

### Imaging and data analysis

For gradient characterization, the green fluorescence of the fluorescein of all 3 channels was recorded to calibrate the gradient profile in the device. The fluorescence was detected using a 4X objective (N.A. 1.3; Olympus, PA) with a 480±40 nm excitation filter, 535±50 nm emission filter, and a 505 nm long-pass dichroic mirror (Chroma Technology Corp., Rockingham, VT) with light from a 100 w mercury lamp. For the first 6 min, the time-lapse images of fluorescence were taken by a SensiCam EM high performance camera (The Cooke Corporation, MI) at 1 fps controlled by IPLab Spectrum software (Version 4.0; Signal Analytics, VA). Afterward, 1 image was taken at 25, 55, 80, 110 and 140 min after t = 0. Data were analyzed by using NIH ImageJ (http://rsbweb.nih.gov/ij/).

For live cell imaging, we used 300X brightfield optics (Carl Zeiss Inc., MA). For high cell density sea urchin sperm migration experiment, a video camera (CCD 72, DAGE MTI, Michigan City, IN) was used to record at 30 fps for experiments lasting for minutes (Maxwell Professional SVHS, Osaka, Japan; SR-S365U SuperVHS Video Cassette Recorder, JVC, Long Beach, CA). For all the other experiments, a Redlake MotionPro high speed digital camera (IDT, Tallahassee, FL) was used to record at 50 fps for 38 sec and the video was post-processed by the MiDAS 2.0 software.

We tracked the trajectories of the centroids of the heads of sperm through the time series images using a manual tracker tool in ImageJ. Only cells with beating flagella were tracked. To avoid wall boundary effects, we tracked cells located in the middle 200 µm out of the 400 µm channel width for mouse sperm; and cells located in the middle 300 µm out of the 400 µm channel width for sea urchin sperm (See Fig. S1).

Cell motility was quantified using the average cell speed *U*, defined as the length that the cell traveled within a specific track divided by elapsed time. Cell chemotaxis was quantified by the average cell velocity *V_x_* along the gradient direction (x-axis), defined as the net displacement along the gradient direction within each track divided by time. We also computed persistence length, defined as the net displacement of the cell for each track divided by the length of the track. All parameters were computed using a custom Matlab code (The MathWorks, Inc., Natick, MA). The average speed, velocity and persistence length were computed from about 30 or more cell tracks under the same chemical condition, which came from three separate experiments. Using Prism 5 software (GraphPad Software, Inc., La Jolla, CA), mean values were calculated and statistical tests were conducted using a one-way ANOVA method and *t*-test between treatment and control groups.

## Supporting Information

Figure S1 **Drawing for the simulation model.** The dimension of the computation frame was 10 mm wide along *x*′ axis and 1 mm high along *z*′ axis and the channel size was 400 μm wide and 167 μm high with a 250 μm gap. The dashed line indicates the middle plane of the center channel where sperm motion was imaged, which is 83 μm from the bottom. Please note that the assessed section in Fig. S2 is the entire center channel (400 μm), while those in Fig. S3 are central portions of the center channel (300 μm for sea urchin and 200 μm for mouse sperm).
(TIF)

Figure S2 **Comparison of gradient establishment between experiments (A) and computer simulation (B).** The experimental results were obtained by re-plotting fluorescence of FITC-dextran (4 kDa) from Fig. 1. The simulation results were evaluated along with the same channel area at a half of the channel height (*z*′ = 83 μm, or dashed line in Fig. S1). Each colored line represents concentration profile at a given time. The time is coded in the color, as shown on the right side of the figure. The stable gradient establishing time is around ∼25 min in both cases.
(TIF)

Figure S3 **Concentration profiles across the area of interests in the center channel during the experiment time period.** A and B: The time evolution of resact concentration profiles in the first 38 s across the middle section (300 µm) of the center channel where the sea urchin sperm motion was analyzed. The time interval between lines was 2 s. The perfused concentration of resact in the source channel was (A) 100 pM and (B) 10 nM. C and D: The time evolution of progesterone concentration in the first 14 s over the middle section (200 µm) of the center channel where the mouse sperm motion was analyzed. The time interval between lines is 1 s. The perfused concentration of progesterone in the source channel was (C) 2.5 µM and (D) 250 µM.
(TIF)

Movie S1 **Sea urchin sperm swimming in a microfluidic channel in the absence of resact (Control).** Sperm seeding density is 8×10^6^ /ml. Each image has a size of 400 µm ×200 µm. The video is played in real time at 25 fps. Duration of the video is 19 s.
(AVI)

Movie S2 **Mouse sperm swimming in a microfluidic channel in the absence of progesterone.** Sperm seeding density is 10×10^6^ /ml. Each image has a size of 400 µm ×200 µm. The video is played in real time at 25 fps. Duration of the video is 19 s.
(AVI)

Movie S3 **Sea urchin sperm swimming in the center channel when 10 nM reseact solution and medium were pumped through the source and sink channels, respectively, starting at t = 0.** Sperm seeding density is 8×10^6^ /ml. Each image has a size of 400 µm ×200 µm. The video is played in real time at 25 fps. The resact is diffusing from left to the right in the central channel. Duration of the video is 19 s.
(AVI)

Movie S4 **Mouse sperm swimming in the center channel when 250 µM progesterone and medium were pumped through the source and sink channels, respectively, starting at time 0.** Sperm seeding density is 10×10^6^ /ml. Each image has a size of 400 µm ×200 µm. The video is played in real time at 25 fps. The progesterone is diffusing from the left to the right side of the central channel. Duration of the video is 19 s.
(AVI)

Movie S5 **Sea urchin sperm swimming in center channel when 250 nM resact and medium were pumped through the source and sink channels, respectively, starting at time 0.** Sperm seeding density is 120×10^6^/ml. Each image has a size of 400 µm ×200 µm. The video plays at 10 fps and 5 times faster than real time. Initially, the resact diffused from the right to the left side of the central channel; then the two tubes were switched so the resact started to diffuse from the left to the right. Duration of the video is 33 s.
(AVI)

Text S1 **The text describes the detailed work of the simulation of concentration profiles.**
(DOCX)
