# Supplementary figures and images for: Different Migration Patterns of Sea Urchin and Mouse Sperm Revealed by a Microfluidic Chemotaxis Device
Source: PLoS One. 2013 Apr 16;8(4):e60587. doi: 10.1371/journal.pone.0060587 (PMC3628882; doi:10.1371/journal.pone.0060587)

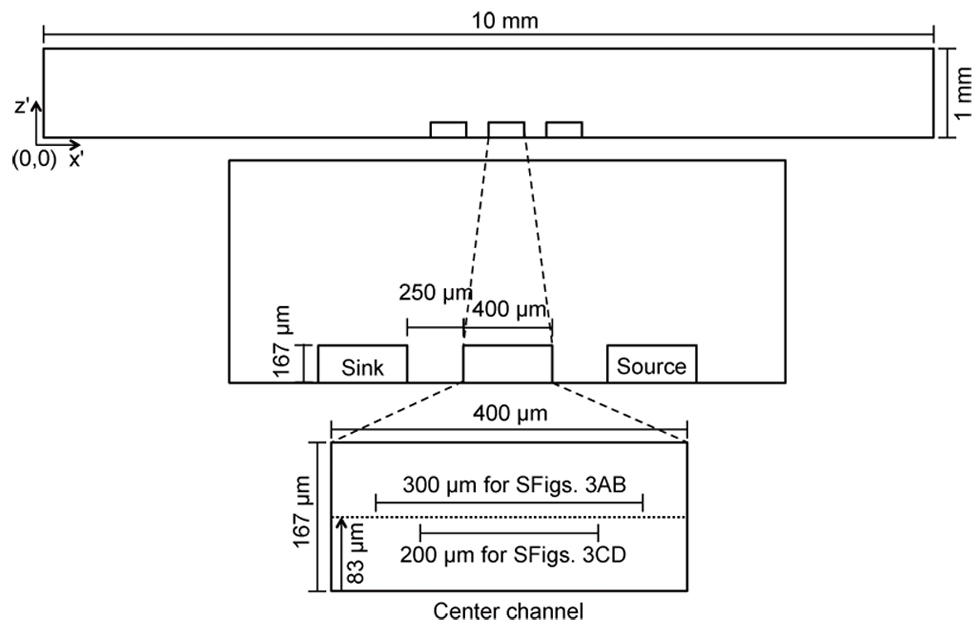

Supplement: Figure S1 — Drawing for the simulation model. The dimension of the computation frame was 10 mm wide along x′ axis and 1 mm high along z′ axis and the channel size was 400 μm wide and 167 μm high with a 250 μm gap. The dashed line indicates the middle plane of the center channel where sperm motion was imaged, which is 83 μm from the bottom. Please note that the assessed section in Fig. S2 is the entire center channel (400 μm), while those in Fig. S3 are central portions of the center channel (300 μm for sea urchin and 200 μm for mouse sperm). (TIF) [file pone.0060587.s001.tif]

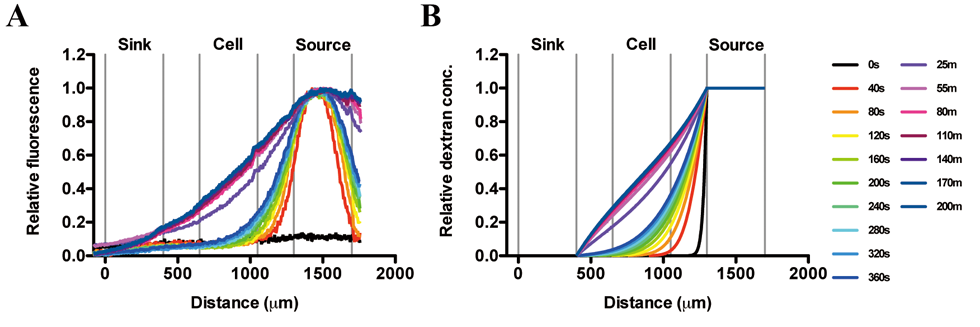

Supplement: Figure S2 — Comparison of gradient establishment between experiments (A) and computer simulation (B). The experimental results were obtained by re-plotting fluorescence of FITC-dextran (4 kDa) from Fig. 1. The simulation results were evaluated along with the same channel area at a half of the channel height (z′ = 83 μm, or dashed line in Fig. S1). Each colored line represents concentration profile at a given time. The time is coded in the color, as shown on the right side of the figure. The stable gradient establishing time is around ∼25 min in both cases. (TIF) [file pone.0060587.s002.tif]

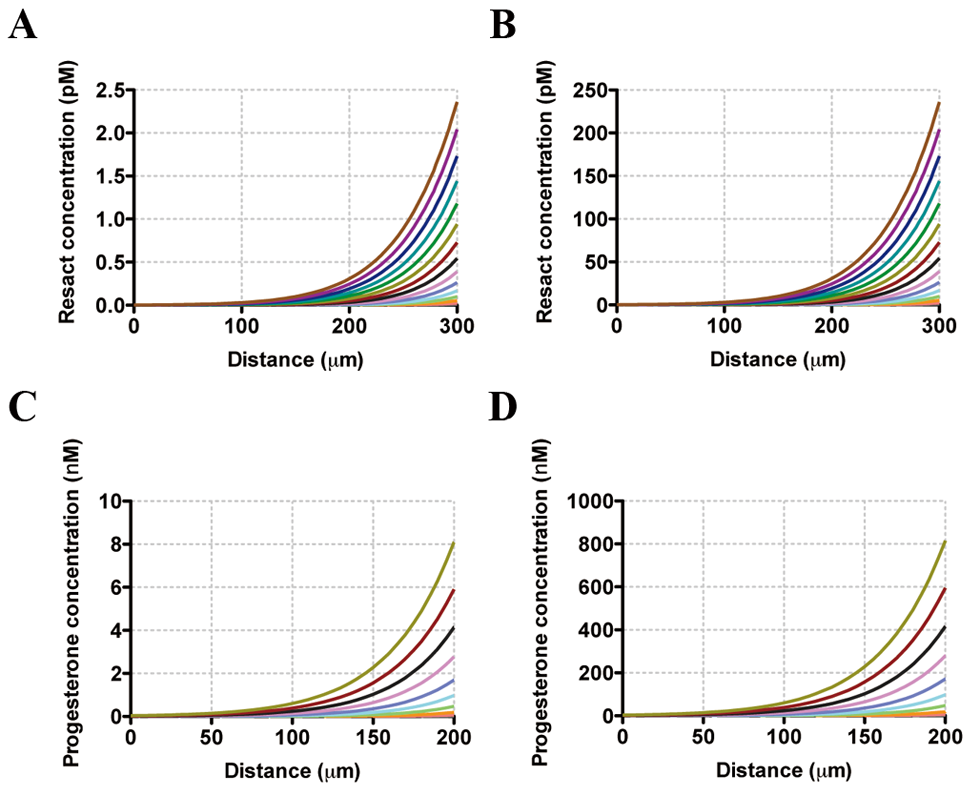

Supplement: Figure S3 — Concentration profiles across the area of interests in the center channel during the experiment time period. A and B: The time evolution of resact concentration profiles in the first 38 s across the middle section (300 µm) of the center channel where the sea urchin sperm motion was analyzed. The time interval between lines was 2 s. The perfused concentration of resact in the source channel was (A) 100 pM and (B) 10 nM. C and D: The time evolution of progesterone concentration in the first 14 s over the middle section (200 µm) of the center channel where the mouse sperm motion was analyzed. The time interval between lines is 1 s. The perfused concentration of progesterone in the source channel was (C) 2.5 µM and (D) 250 µM. (TIF) [file pone.0060587.s003.tif]
