# Supplementary material for: Different Migration Patterns of Sea Urchin and Mouse Sperm Revealed by a Microfluidic Chemotaxis Device
Source: PLoS One. 2013 Apr 16;8(4):e60587. doi: 10.1371/journal.pone.0060587 (PMC3628882; doi:10.1371/journal.pone.0060587)
Supplement: Text S1 — The text describes the detailed work of the simulation of concentration profiles. (DOCX) [file pone.0060587.s009.docx]

**SUPPORTING INFORMATION TEXT**

**Simulation of concentration profiles**

We verified and calculated concentration profiles of resact and progesterone during the time period of the experiment, using the COMSOL Multiphysics software (COMSOL, Inc., Burlington, MA) (1, 2). In our calculation, we used diffusion coefficients of 25 and 52.1×10^-7^ cm^2^/s for resact (m.w. = 1,245 Da) and progesterone (m.w. = 314 Da), respectively (3, 4). The model was built with a built-in time-dependent 2D diffusion model with a dilute solute. The dimension of the computation frame was 10 mm wide and 1 mm high (See Fig. S1). The sizes of the three channels were chosen to be exactly the same as in the experimental setting, which was 400 μm wide and 167 μm high with a 250 μm gap (see Fig. S1). The initial condition was $C_{t=0}=0$ everywhere, except the source channel, where the concentration was 100 pM or 10 nM for resact and 2.5 or 250 μM for progesterone. No flux boundary conditions were chosen at the horizontal ends $\left. \frac{\partial C}{\partial x^{'}} \right|_{x^{'}=0,10mm}=0$, and at the vertical ends $\left. \frac{\partial C}{\partial z^{'}} \right|_{z^{'}=0,1mm}=0$. Since the imaging plane of the sperm motion was at the mid-plane where z’ = 83 μm (See SFig. 1), all the computed concentration profiles shown below were taken in this plane. We meshed the entire area with a maximum element size of 10 μm, a minimum of 0.1 μm, a maximum element growth rate of 1.1, a resolution of curvature of 0.2, and a resolution of narrow regions of 1. The resulting complete mesh consisted of 257,604 elements.

To verify the computation results, we first computed concentration profiles of FITC-dextran (m.w. = 4 kDa) across all three channels at various time points together with those measured using fluorescence (See Fig. S2). The simulated time interval was 2 seconds. Similar characteristics are shown in Figs. S2*A* and S2*B*. Furthermore, the time at which the concentration profiles reach a steady state is about 25 minutes. This is consistent with the estimated value of gradient establishing time of ~ 20 min, where *l* = 900 μm is the distance from source to sink channel and $t=l^{2}/4D$. Fig. S2*A* is generated by replotting Fig. 1 with relative fluorescence. Fig. S2*B* is obtained with a diffusion coefficient of 17×10^-7^ cm^2^/s and 1 μM dextran (4 kDa) (3).

To evaluate the concentration profiles for our experiments, we first computed the concentration profiles of resact over the middle section of the center channel (300 μm) at $z^{'}$ = 83 μm (a half of the channel height) for 38 s (See Fig. S1). The simulated time interval was 0.1 seconds. It should be noted that 38 s is the longest duration that sea urchin sperm cells stay in the middle section of the center channel before reaching at either right or left sides 50-μm away from the wall. As seen in Fig. S3*A*, the concentration profiles evolves with time rapidly during this time period. The maximum concentration in the area of interest was found to be 2.4 pM or 240 pM for the case where 100 pM or 10 nM resact was perfused in the source channel respectively (Figs. S3*AB*). We then computed the concentration profiles of progesterone over the middle section of the center channel (200 μm) for 14 s as shown in Fig. S3*B*. It should be noted that 14 s was the maximum duration time that the mouse sperm cells stay in the center portion of the center channel (See Fig. S1) before reaching at either ends of 200 μm section (100 μm away from the wall). As seen in Figs. S3*CD*, the concentration profiles evolve with time rapidly. A maximum of 8.1 or 820 nM of progesterone concentration was obtained for the case where 2.5 or 250 μM of progesterone flows in the source channel respectively (Figs. S3*CD*).

1. Haessler U, Pisano M, Wu M, Swartz MA (2011) Dendritic cell chemotaxis in 3D under defined chemokine gradients reveals differential response to CCL21 and CCL19. *Proc Natl Acad Sci USA* 108: 5614-5619.

2. Haessler U, Kalinin Y, Swartz MA, Wu M (2009) An agarose-based microfluidic platform with a gradient buffer for 3D chemotaxis studies. *Biomed Microdevices* 11:827-835.

3. Polson A (1950) Some aspects of diffusion in solution and a definition of a colloidal particle. *J Phys Chem* 54:649-652.

4. Lebrun L, Junter GA (1993) Diffusion of sucrose and dextran through agar gel membranes. *Enzyme Microb Technol* 15:1057-1062.
